# Supplementary material for: Content-rich biological network constructed by mining PubMed abstracts
Source: BMC Bioinformatics. 2004 Oct 8;5:147. doi: 10.1186/1471-2105-5-147 (PMC528731; doi:10.1186/1471-2105-5-147)
Supplement: Additional File 2 — The original results of the above study (non-essential files are deleted to keep the file size under the limit set by BMC bioinformatics). [file 1471-2105-5-147-S2.bz2 › chilibotAdditionalFile2/dip30/467ID9195914E10495/html/IL8RB_CXCL1.html]

 


 **IL8RB** and **CXCL1** 
  
Found 79 abstracts in PubMed, retrieved 30.  
 

 What does Google say? 
 PDF only 
| .edu only 

---

**Interactive relationship** (e.g. stimulation, inhibition, etc)

**Inhibitory relationship**- Our data suggests that the anti apoptotic effect of Gro  [ **CXCL1** ]  alpha is mediated through CXCR2  [ **IL8RB** ]  as selective CXCR2  [ **IL8RB** ]  blockade with SB272844 can potently abrogate this response.  Ref: 12090783 Pulm Pharmacol Ther, 2002
- CXCL10 inhibits  **CXCL1**  induced PAK1 activation and chemotaxis by interfering with appropriate binding of  **CXCL1**  to CXCR2  [ **IL8RB** ]  receptor.  Ref: 12549928 Biochemistry, 2003
- Selective CXCR2  [ **IL8RB** ]  antagonists may be useful to block the Gro  [ **CXCL1** ]  alpha induced priming response whilst preserving neutrophil functions mediated by CXCR1, the alternative neutrophil receptor for interleukin 8.  Ref: 11589350 Eur Respir J, 2001
- The pro survival effect of a fixed concentration of agonist IL 8 or Gro  [ **CXCL1** ]  alpha on cultured neutrophils was abrogated by a selective CXCR2  [ **IL8RB** ]  antagonist SB272844 K D s 253 nM and 49.9 nM in the presence of IL 8 or Gro  [ **CXCL1** ]  alpha respectively.  Ref: 12090783 Pulm Pharmacol Ther, 2002
**Neutral relationship**- The GRO  [ **CXCL1** ]  alpha receptor CXCR2  [ **IL8RB** ]  was also detected in tumor tissue, proposing interactions between eosinophils and the tumor.  Ref: 12734381 J Immunol, 2003
- Characterization of the molecular interactions of interleukin 8 CXCL8, growth related oncogen alpha  **CXCL1**  and a non peptide antagonist SB 225002 with the human CXCR2  [ **IL8RB** ] .  Ref: 12628493 Biochem Pharmacol, 2003
- Growth regulated oncogene alpha GRO  [ **CXCL1** ]  alpha in interaction with its receptor CXCR2  [ **IL8RB** ]  is a chemoattractant for both T cells and neutrophils and may be one of the chemokines involved in the cell maintenance.  Ref: 11524231 J Endocrinol, 2001
- Together these experiments support the notion that increased expression of KC  **CXCL1**  and MIP 2 CXCL2 3 and their interaction with CXCR2  [ **IL8RB** ]  are important in the pathogeneses of VILI.  Ref: 12464676 J Clin Invest, 2002
- These data indicate that HSPGs are involved in modulating  **CXCL1**  induced PAK1 activation and chemotaxis through regulating  **CXCL1**  binding activity to CXCR2  [ **IL8RB** ]  receptor.  Ref: 12549928 Biochemistry, 2003
- It is shown here that  **CXCL1**  induces cdc42 and PAK1 activation in CXCR2  [ **IL8RB** ]  expressing HEK293 cells.  Ref: 12033944 Biochemistry, 2002
- In addition, Gro  [ **CXCL1** ]  alpha was chemotactic for T cells expressing CXCR2  [ **IL8RB** ]  in vitro toward both recombinant protein and tumor derived chemokine.  Ref: 12427307 Hum Gene Ther, 2002
- ... several chemokines, including growth related oncogen alpha  **CXCL1**  and a non peptide antagonist SB 225002 are specific for CXCR2  [ **IL8RB** ] .  Ref: 12628493 Biochem Pharmacol, 2003
- CXCR1 and CXCR2  [ **IL8RB** ]  are receptors for ELR positive CXC chemokines which include growth regulated oncogene alpha GRO  [ **CXCL1** ]  alpha and interleukin 8 IL 8.  Ref: 11356265 Brain Res Dev Brain Res, 2001
- CXCR2  [ **IL8RB** ]  binds IL 8 and other structurally related chemokines such as growth related oncogene GRO  [ **CXCL1** ]  a, GRObeta, GROgamma, neutrophil activating peptide 2 NAP 2 and epithelial cell derived neutrophil activating peptide 78 ENA 78.  Ref: 12365735 Fish Shellfish Immunol, 2002
- Interestingly we demonstrate, for the first time, that Gro  [ **CXCL1** ]  alpha was able to induce interferon gamma IFN gamma secretion from transduced T cells, thereby extending our knowledge of other potential functions of CXCR2  [ **IL8RB** ] .  Ref: 12427307 Hum Gene Ther, 2002
- Whole lung levels of IFN gamma, inflammatory protein 10 CXC ligand CXCL  [ **CXCL1** ]  10, and monokine induced by IFN gamma MIG CXCL9 were significantly increased in CXCR2  [ **IL8RB** ]  mice compared with CXCR2  [ **IL8RB** ]  mice at various times after conidia.  Ref: 11801688 J Immunol, 2002

**Non-interactive relationship** (e.g. studied together, co-existance, homology, etc.)

- Furthermore we demonstrate that both CG 4 cells and primary cultures of rat OLPs are immunoreactive for CXCR2  [ **IL8RB** ] , the potential receptor for GRO  [ **CXCL1** ]  alpha.  Ref: 11356265 Brain Res Dev Brain Res, 2001
- We show here that the  **CXCL1**  maximal binding to CXCR2  [ **IL8RB** ]  expressed on HEK293 and CHO K1 cells is dependent on the presence of cell surface HSPGs.  Ref: 12549928 Biochemistry, 2003
- In addition, we demonstrate that compounds which inhibit the actions of IL 8 and GRO  [ **CXCL1** ]  alpha via their receptors, CXCR1 and CXCR2  [ **IL8RB** ] , also inhibit HIV 1 replication in both T lymphocytes and macrophages, indicating potential therapeutic uses for these compounds in HIV 1 infection and AIDS.  Ref: 11483765 J Virol, 2001
- GRO  [ **CXCL1** ]  alpha then fed back and stimulated HIV 1 replication in both MDM and lymphocytes, and antibodies that neutralize GRO  [ **CXCL1** ]  alpha or CXCR2  [ **IL8RB** ]  the receptor for GRO  [ **CXCL1** ]  alpha markedly reduced viral replication in MDM and peripheral blood mononuclear cells.  Ref: 11390582 J Virol, 2001
- The aims of this study were to investigate the effects of Interleukin 8 IL 8 and Growth related oncogene alpha Gro  [ **CXCL1** ]  alpha on neutrophil apoptosis and determine the potential for a selective CXCR2  [ **IL8RB** ]  antagonist to inhibit these responses.  Ref: 12090783 Pulm Pharmacol Ther, 2002
- For example strong calcium responses were seen in neutrophils following stimulation with the CXCR1 and CXCR2  [ **IL8RB** ]  ligands, interleukin IL 8, GCP 2 and Gro  [ **CXCL1** ]  beta.  Ref: 11298490 Cytokine, 2001
- Analysis by reverse transcriptase polymerase chain reaction and enzyme linked immunosorbent assay showed CXCR2  [ **IL8RB** ]  messenger ribonucleic acid and GRO1  [ **CXCL1** ]  protein expression were present in oligodendrogliomas.  Ref: 11322447 Neurosurgery, 2001
- To better understand the molecular mechanisms by which  **CXCL1**  induces CXCR2  [ **IL8RB** ]  mediated chemotaxis, the signal transduction components involved in  **CXCL1**  induced chemotaxis were examined.  Ref: 12033944 Biochemistry, 2002
- However, T cells did not express the receptor for Gro  [ **CXCL1** ]  alpha, CXCR2  [ **IL8RB** ] , and therefore, T cells were transduced with a retroviral vector encoding CXCR2  [ **IL8RB** ] .  Ref: 12427307 Hum Gene Ther, 2002
- In slice preparations,  **CXCL1**  inhibited embryonic oligodendrocyte precursor migration, and widespread dispersal of postnatal precursors occurred in the absence of CXCR2  [ **IL8RB** ]  signaling.  Ref: 12176324 Cell, 2002
- Pretreatment of these cells with anti CXCR2  [ **IL8RB** ]  did not inhibit the chemotactic response to GRO  [ **CXCL1** ]  alpha.  Ref: 11154210 Blood, 2001
- The percentage of PBNs to undergo shape change %SC, spontaneously and in response to IL 8 and Gro  [ **CXCL1** ]  alpha, was measured in patients with CFA n=10 and controls n=10, and the effect of the CXCR2  [ **IL8RB** ]  antagonist SB272844 studied.  Ref: 11589350 Eur Respir J, 2001
- b for  **CXCL1** , the N terminus of CXCR2  [ **IL8RB** ]  is necessary but not sufficient for binding.  Ref: 12628493 Biochem Pharmacol, 2003
- Here we demonstrate a role for the chemokine  **CXCL1**  and its receptor CXCR2  [ **IL8RB** ]  in patterning the developing spinal cord.  Ref: 12176324 Cell, 2002
- PMNs were exposed to interleukin 8 IL 8, growth regulated oncogene alpha GRO  [ **CXCL1** ]  alpha, and platelet activating factor PAF to stimulate the CXCR1, CXCR2  [ **IL8RB** ] , and PAF receptors.  Ref: 11130495 J Trauma, 2000
- In summary, we have shown that TII alveolar epithelial cells produce three of the major proinflammatory CXC chemokines, GRO  [ **CXCL1** ] , CINC 2alpha and MIP 2, and their cognate receptor CXCR2  [ **IL8RB** ] .  Ref: 12829448 Am J Respir Cell Mol Biol< MedlineTA>Am J Respir Cell Mol Biol, 2000
- Because the murine CXC chemokines KC  **CXCL1**  and MIP 2 CXCL2 3, via CXCR2  [ **IL8RB** ] , are potent neutrophil chemoattractants, we investigated their role in a murine model of VILI.  Ref: 12464676 J Clin Invest, 2002
- Firm adhesion and sequestration of monocytes on activated MC was supported by the GRO  [ **CXCL1** ]  alpha receptor CXCR2  [ **IL8RB** ]  and to a lesser extent by CX 3 CR.  Ref: 11313419 J Immunol, 2001
- This study investigated whether, in CFA, the PBN motility response is primed by IL 8 and growth related oncogene Gro  [ **CXCL1** ]  alpha, as demonstrated in other neutrophilic inflammatory diseases, and whether the motility response of PBNs to IL 8 and Gro  [ **CXCL1** ]  alpha can be abrogated using a selective antagonist for the neutrophil receptor for IL 8 and Gro  [ **CXCL1** ]  alpha, CXCR2  [ **IL8RB** ] .  Ref: 11589350 Eur Respir J, 2001
- Signaling through CXCR2  [ **IL8RB** ] ,  **CXCL1**  inhibited oligodendrocyte precursor migration.  Ref: 12176324 Cell, 2002
- Finally, the mechanism of binding of a non peptide antagonist on CXCR2  [ **IL8RB** ]  was investigated it occurred through epitopes a which were disseminated within the receptor, b which differed according to the use of CXCL8 or  **CXCL1**  as a competitor and c which did not necessarily overlap with agonist binding sites.  Ref: 12628493 Biochem Pharmacol, 2003
- The chemotactic response to the CXC and CC chemokines correlated with the receptor expression except that all 3 populations responded to GRO  [ **CXCL1** ]  alpha, despite their lack of CXCR2  [ **IL8RB** ]  expression.  Ref: 11154210 Blood, 2001
- RESULTS Immunohistochemistry demonstrated that all components of the GRO1  [ **CXCL1** ]  PDGF pathway GRO1  [ **CXCL1** ]  protein, its receptor CXCR2  [ **IL8RB** ] , PDGF A chain, and its receptor PDGFalphaR were expressed by tumor cells in 6 86% of 7 of oligodendrogliomas as well as by 0 of 4 diffuse astrocytomas World Health Organization Grades II and III and 2 18% of 11 glioblastomas.  Ref: 11322447 Neurosurgery, 2001
